# Multifactorial falls prevention programme compared with usual care in UK care homes for older people: multicentre cluster randomised controlled trial with economic evaluation

**DOI:** 10.1136/bmj-2021-066991

**Published:** 2021-12-07

**Authors:** Pip A Logan, Jane C Horne, John R F Gladman, Adam L Gordon, Tracey Sach, Allan Clark, Katie Robinson, Sarah Armstrong, Sue Stirling, Paul Leighton, Janet Darby, Fran Allen, Lisa Irvine, Ed C F Wilson, Chris Fox, Simon Conroy, Gail Mountain, Karen McCartney, Maureen Godfrey, Erika Sims

**Affiliations:** 1 School of Medicine, Queens Medical Centre, University of Nottingham, Nottingham, NG7 2UH, UK; 2 Nottingham CityCare Partnership, Nottingham, UK; 3 NIHR Applied Research Collaboration – East Midlands, Nottingham, UK; 4 NIHR Nottingham Biomedical Research Centre, Nottingham, UK; 5 Nottingham University Hospitals NHS Trust, Nottingham, UK; 6 University Hospitals of Derby and Burton NHS Foundation Trust, Derby, UK; 7 Norwich Medical School, University of East Anglia, Norwich, UK; 8 University of Hertfordshire, Hatfield, UK; 9 Exeter Medical School, University of Exeter, Exeter, UK; 10 University College London, London, UK; 11 University of Bradford, Bradford, UK; 12 Nottingham, UK

## Abstract

**Objectives:**

To determine the clinical and cost effectiveness of a multifactorial fall prevention programme compared with usual care in long term care homes.

**Design:**

Multicentre, parallel, cluster randomised controlled trial.

**Setting:**

Long term care homes in the UK, registered to care for older people or those with dementia.

**Participants:**

1657 consenting residents and 84 care homes. 39 were randomised to the intervention group and 45 were randomised to usual care.

**Interventions:**

Guide to Action for Care Homes (GtACH): a multifactorial fall prevention programme or usual care.

**Main outcome measures:**

Primary outcome measure was fall rate at 91-180 days after randomisation. The economic evaluation measured health related quality of life using quality adjusted life years (QALYs) derived from the five domain five level version of the EuroQoL index (EQ-5D-5L) or proxy version (EQ-5D-5L-P) and the Dementia Quality of Life utility measure (DEMQOL-U), which were self-completed by competent residents and by a care home staff member proxy (DEMQOL-P-U) for all residents (in case the ability to complete changed during the study) until 12 months after randomisation. Secondary outcome measures were falls at 1-90, 181-270, and 271-360 days after randomisation, Barthel index score, and the Physical Activity Measure-Residential Care Homes (PAM-RC) score at 91, 180, 270, and 360 days after randomisation.

**Results:**

Mean age of residents was 85 years. 32% were men. GtACH training was delivered to 1051/1480 staff (71%). Primary outcome data were available for 630 participants in the GtACH group and 712 in the usual care group. The unadjusted incidence rate ratio for falls between 91 and 180 days was 0.57 (95% confidence interval 0.45 to 0.71, P<0.001) in favour of the GtACH programme (GtACH: six falls/1000 residents *v* usual care: 10 falls/1000). Barthel activities of daily living indices and PAM-RC scores were similar between groups at all time points. The incremental cost was £108 (95% confidence interval −£271.06 to 487.58), incremental QALYs gained for EQ-5D-5L-P was 0.024 (95% confidence interval 0.004 to 0.044) and for DEMQOL-P-U was 0.005 (−0.019 to 0.03). The incremental costs per EQ-5D-5L-P and DEMQOL-P-U based QALY were £4544 and £20 889, respectively.

**Conclusions:**

The GtACH programme was associated with a reduction in fall rate and cost effectiveness, without a decrease in activity or increase in dependency.

**Trial registration:**

ISRCTN34353836.

## Introduction

Falls are three times more common in care home residents than people of similar age living in the community.^1^ In the United Kingdom, around 4% of those older than 65 years and 15% of those older than 85 years live in care homes that provide 24 hour care with or without nursing input.^2^ Falls are a problem in the current population of 400 000 care home residents living in the UK, most of whom are at high risk of falling. This poses a high cost to society and adds burden to health and social care systems, such as ambulance services and emergency departments. Falls are associated with personal cost to residents and their families, with some residents never returning to their previous level of function. Most residents have multiple medical conditions and limitations in activities of daily living.^3^ Serious injuries account for around 25% of falls in care home residents, and 40% of admissions from care home to hospital are related to falls.^4^ ^5^ A systematic review of the randomised controlled trial evidence in 2018 concluded that effective interventions to prevent falls in community dwelling people (exercise, drug review, and multifactorial interventions) were of uncertain benefit in care home residents.^6^ Given the multiple intrinsic and extrinsic risk factors for falls in care homes, the review advised further evaluation of multifactorial interventions.

Using a co-design approach, our research group developed and tested in a feasibility randomised controlled trial, an intervention programme to prevent falls in care home residents, the Guide to Action Care Home (GtACH) programme.^7^ ^8^ These reported results provided the evidence and justification to conduct a full definitive trial. The multi-domain GtACH programme includes one hour of training for all care home staff (including gardeners, caretakers, cooks, cleaners, managers) in small groups, delivered by a falls specialist. After training, a manual summarising the GtACH programme and including resources such as a falls incident chart (to detect patterns) and a drug falls risk chart is left in the care home. Once trained, staff are expected to use the GtACH risk assessment and checklist for all residents. For example, the assessment might highlight that a resident is dehydrated, and the recommended action is to increase fluid intake. The manual and training prompts care home staff to take action for the dehydration, such as introducing smoothies, offering fruit juice more often, adapting crockery to take account of disabilities, producing soups, and making an event of coffee time. Overall, the training and resources increase both awareness and knowledge about the management of falls. In our multicentre randomised controlled trial (the Falls in Care Homes (FinCH) study), we determined the clinical and cost effectiveness of the GtACH programme compared with usual care in UK care homes.

## Methods

We performed a multicentre, parallel, 1:1 cluster randomised controlled trial to evaluate the GtACH programme compared with usual care for the prevention of falls in older residents of long term care homes in the UK; the full protocol has been published.^9^ The primary health outcome was fall rate 91-180 days after randomisation of the care home. This timeframe was chosen to allow time to train and embed the use of the programme, as determined by the feasibility study.^8^ A within trial economic evaluation estimated the cost effectiveness of the GtACH programme from a health and personal social services perspective, as recommended.^10^ This trial was designed as near to real life as possible, such that the percentage of care home staff trained to use the GtACH programme in the intervention homes was collected but the number of actions to prevent falls, such as new spectacles, was not collected. Our process evaluation completed in six homes will be presented in a separate paper.

### Care home and participant recruitment

Sites comprised National Health Service organisations with established community based falls teams that were willing to participate in the trial, and the care homes in geographical areas covered by the corresponding NHS Clinical Commissioning Group or Integrated Care System. Sites were identified through the National Institute of Health Research Clinical Research Network, which aims to connect research teams with relevant clinical services. Ten sites across England recruited eligible care homes. To be eligible the homes had to be long stay and registered to care for older people or those with dementia, have 10 or more residents, routinely record falls, and have the agreement of the care home owner. Care homes were not eligible if they had participated in previous GtACH studies,^8^ provided care for those with learning difficulties, did not agree to the intervention being used, or were under special measures from the UK national regulator of care homes (the Care Quality Commission). Most care homes in the UK are privately owned and none are provided by the NHS. They are paid for by residents, or by the state if residents are unable to pay. The NHS provide healthcare to residents as if they were living in their own homes—for example, each resident is registered with a local general practitioner. Owners of care homes employ a range of professionally registered healthcare workers, such as nurses and unregistered care staff, depending on their licence.

Care homes were identified from the register of care homes held by the Care Quality Commission; contacted by email, telephone, and letter; and recruited by research staff in person. At this meeting the researcher explained the study, ensured that falls were being recorded in the personal care records and on the falls incident sheets according to the recommendations of the Care Quality Commission, and used the same definition of a fall—an unintentional or unexpected loss of balance resulting in coming to rest on the floor, the ground, or an object below knee level.^10^

All residents in the recruited homes were eligible to participate, including those who lacked mental capacity to provide consent, except for residents who the care home staff determined to be in the last few days of life or who were receiving short term care or rehabilitation. A member of the research team approached and recruited residents with mental capacity. Residents lacking mental capacity were recruited through personal (family or friends) or professional (care home manager) consultees. The time between the first participant recruited and randomisation was four weeks.

### Randomisation and blinding to allocation

Randomisation took place four weeks after recruitment of the first resident in a participating home, as our previous feasibility showed that little additional recruitment was possible after this time. Site trial coordinators randomised care homes on a 1:1 basis to one of two parallel arms (the GtACH programme or usual care) using a bespoke computer generated pseudo-random code of variable block randomisation within strata (site, care home type (nursing, residential, dual registration)) provided by the Norwich Clinical Trials Unit through a secure web based randomisation service. Control homes were offered the intervention at the end of the trial. The researchers, resident participants, and staff informants were blind to allocation at consent and to baseline data collection. Researchers collecting data remained blind to allocation but documented if they became unblinded. By the nature of the intervention, care home staff and resident participants could not be blind to allocation group. All Hospital Episode Statistics data were extracted and analysed blind to allocation. The data monitoring committee were not blinded to the allocation for safety events. Treatment allocations were concealed from the study statistician until the main analyses were complete.

### Intervention

The published GtACH programme^7^ ^8^ is described in full detail using the headings from the Template for Intervention Description and Replication (TIDiER) checklist and a full TIDiER checklist in a supplementary file (supplementary materials S1).^11^

#### Rationale

Owing to care home staff being relatively untrained, the complex nature of risk factors for falls in care residents, and the need for several interventions to deal with multiple risk factors, a systematic care home wide programme including staff education and support in the use of risk assessment and decision support tools was required. The GtACH programme is a systematic approach developed using literature, clinical expertise, and the views of care home residents and families, care home staff, and researchers. The theory was that staff are key to reducing fall rate in care facilities and that by numerous incremental actions, such as improved lighting, greater access to appropriate drinks, timely drug reviews, and monitoring the pattern of falls then the effect on an individual will be seen.

#### GtACH programme

A power point presentation was used in the training. Care home staff received a manual, a paper falls screening and assessment tool, a paper Falls Incident Analysis template, a drug and falls chart, training attendance certificate, and poster to remind people to use the GtACH programme.

Falls specialists trained the care home staff in small, one hour group sessions, using case studies and role play to use the GtACH programme in the care homes; repeated training sessions were offered to reach all staff, including managers; and a member of care home staff was allocated to the role of falls champion, responsible for training new staff and embedding the GtACH programme. Once trained, care home staff completed the GtACH risk assessment with every resident and produced a written action plan. This initial assessment took place within four weeks of training, and reassessment was expected to take place every three to six months. The GtACH assessments guided staff to action, which staff were instructed to undertake and record in the resident’s care records. Only residents recruited to the trial were followed up, and therefore the fall rate in non-recruited residents was not available for analysis.

Measures taken to guard against contamination between groups^12^ comprised: explaining the importance of usual care for the control group, training staff in trial design and confidentiality agreements, collating data on staff moving to other homes in the study, and not publishing or sharing the training manual publicly. To aid recruitment, retention, and adherence to the protocol, all control homes were offered the intervention after the 12 month data had been collected and checked.

### Primary outcome

The main outcome measure was the fall rate at 91-180 days after randomisation. Care home staff recorded falls in the resident’s care plans and on incident forms, in keeping with usual standards of care. Every three months, researchers blinded to allocation read all the care plans and recorded the date, place, and impact of falls for all participants, including for those who had died. They cross checked the written care plans with other data held in the care home, such as incident forms, records of ambulance visits, and records of hospital admissions.

### Economic outcomes

The economic evaluation measured health related quality of life using quality adjusted life years (QALYs) derived from the five domain five level version of the EuroQoL index or proxy version (EQ-5D-5L-P)^13^ and the Dementia Quality of Life utility measure (DEMQOL-U),^14^ which were completed by residents who were able to do so and by a member of care home staff as proxy (DEMQOL-P-U) for all residents until 12 months after randomisation. Baseline costs included the GtACH programme and health resource use (primary care, community health, drugs, and social service), and death were identified from care home records. Hospital use and fracture rate were obtained from routine NHS Hospital Episode Statistics reports. We applied unit costs in UK pounds sterling for 2017-18.

### Secondary outcomes

Secondary outcome measures were the rate of falls at 1-90, 181-270, and 271-360 days after randomisation. Outcomes assessed at 3, 6, 9, and 12 months post-randomisation were dependency, assessed by care home staff using the Barthel index,^15^ a 0-20 scale with 20 indicating independence and 0 indicating a need for full care; activity, assessed using the Physical Activity Measure-Residential Care Homes (PAM-RC),^16^ a five item questionnaire scored out of 21, where 21 indicates unrestricted physical activity and mobility and 0 indicates complete immobility; frequency and type of fractures at 1-6 and 7-12 months post-randomisation; and deaths anytime during 12 months post-randomisation.

Adverse events were not recorded during the trial because the GtACH programme was considered a low risk intervention and the feasibility study had not identified specific risks, untoward incidents, or adverse events. Every month the data monitoring and ethical committees compared the fall rates between care homes to check for safety.

### Statistical analysis

Supplementary material S2 provides the full statistical analysis plan. The only deviation from the initial protocol was that to better standardise reporting we relabelled the intervals for falls from months (0-3, 6-9, and 9-12) to days (1-90, 181-270, and 271-360). The sample size was based on the primary randomised controlled trial outcome (fall rate at 91-80 days). The sample size estimate for 1474 residents recruited from 78 care homes was recalculated during the trial because of different observed mean and variability of cluster sizes from anticipated.^9^ The calculation assumed a fall rate of 2.5 falls each year in the control group,^17^ 80% power, a two sided significance level of 5% to detect a 33% reduction in fall rate in the intervention group (as seen in community based falls prevention interventions^6^), mean cluster size 19, coefficient of variation 0.5, and a 16% attrition rate.

Analyses were undertaken on an intention-to-treat basis according to a prespecified statistical analysis plan (available from author). Two sided tests were used, with statistical significance at the 5% level. Baseline characteristics of care homes and residents and outcome measures at baseline and each follow-up time point were summarised by treatment arm using descriptive statistics. The fall rate was expressed as the number of falls per 1000 resident days. We compared the number of falls per resident between groups using a multilevel negative binomial regression model estimated using generalised estimating equations, with care home as the clustering variable. Primary analysis was adjusted for type of care home (residential, nursing, dual registration) and site. An additional model was fitted to assess the robustness of the model. These adjusted for fall rate for the three months before the baseline assessment. Fall rate at 1-90 days, 181-270 days, and 271-360 days were analysed as for the primary outcome. For secondary outcomes, we compared the groups using multilevel regression analysis for continuous outcomes and multilevel logistic regression for binary outcomes. In the secondary outcome analysis, we accounted for clustering by care home using a model with a random intercept for care home in all analyses. A random effect was used to account for clustering by care home. We used multilevel models in all our analyses, with the choice of model depending on the distribution of the outcome measure. Hence the class of modelling was the same for both primary and secondary outcomes, with the only difference being the specific regression model used, which was based on the type of outcome and its distribution. All analyses were prespecified and carried out using STATA 16.1.

Quality of life scores were converted to utilities,^18^ ^19^ from which quality adjusted life years (QALYs) were calculated using linear interpolation and area under the curve analysis with baseline adjustment. If residents died, their utility value and costs were assumed to be zero from the subsequent assessment point and were retained. Costs and outcomes were not discounted, reflecting the timeframe for the analysis. Mean cost and outcomes data were combined to calculate incremental cost effectiveness ratios for both QALY measures, adjusted for age, sex, and site. The cost per fall averted was calculated. Analyses of costs and outcomes used generalised estimating equations regression models. In sensitivity analyses we performed multiple imputation using chained equations using the mi impute command in STATA version 16.^20^ The multiple imputation model included predictors of secondary and non-secondary care costs (baseline and full follow-up); EQ5D-5L and DEMQOL-P based QALYs; and treatment group, care home, age, and sex. The imputation generated values for missing data at each follow-up using ordinary least squares, generating 50 datasets. The generalised estimating equations models were then run on each of these, and the outputs pooled using Rubin’s rules.^21^ This enabled paired cost and outcome data for the entire study population. This was repeated 200 times, with bootstrap replications of the original data. Paired bootstrapped estimates of incremental cost and utility were generated to produce a scatterplot of incremental cost-outcome pairs and the cost effectiveness acceptability curve. All regression analysis was conducted in STATA MP 16^22^ and R.^23^

### Patient and public involvement

A patient and public involvement (PPI) team were instrumental in securing funding, influencing the trial set up, and advocating for care home residents throughout the trial. An adaptation of the research cycle was examined to plan for PPI involvement at each stage of the study, as advocated by UK Standards for Public Involvement.^24^ The aim was to ensure the trial had relevance to care home residents, parties interested in care homes, and the public. The GRIPP2 short form framework^25^ was used to ensure consistency. This approach captures the unique perspective of patients and public experience at each stage of the research cycle. All public facing documents were reviewed by PPI representatives, and PPI members participated as lay researchers in the process analysis. PPI representatives attended all project management meetings. PPI representatives are already working with the research team on a follow-up implementation study, where they are helping us co-design, in conjunction with care home staff representatives, a package to implement the findings from the work presented in this paper. A PPI representative is a co-author on this paper.

## Results

**Fig 1:**
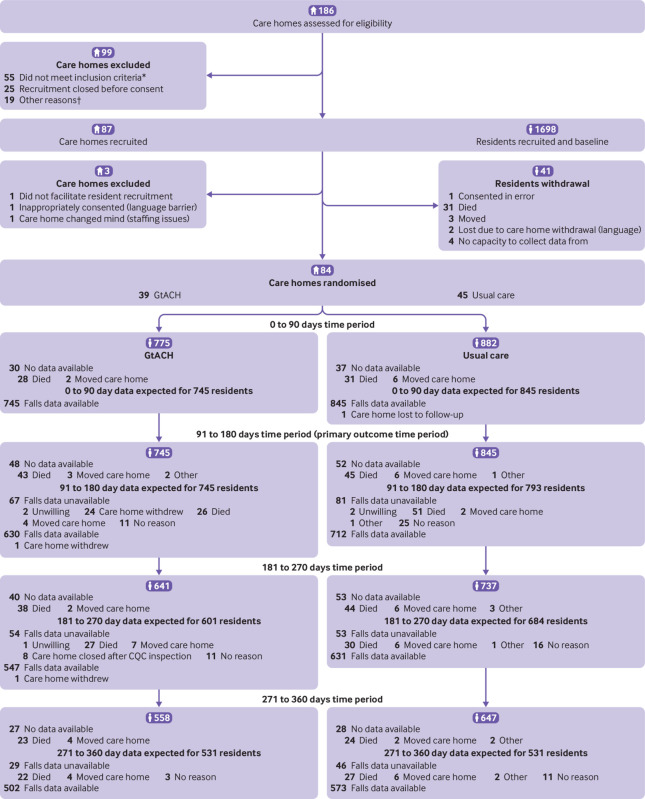
Flow of and care homes and residents through study. *Not prepared to allocate a falls champion (n=24), existing falls programme (n=13), participated in previous studies (n=4), learning disability (n=3), under review (n=1), no reason given (n=10). †Did not have time (n=1), stopped communicating with researcher (n=15), adopted a falls intervention (n=3). Care homes considered lost to follow-up did not respond to multiple requests for follow-up data collection. GtACH=Guide to Action for Care Homes programme; CQC=Care Quality Commission

Recruitment took place between 1 November 2016 and 31 January 2018. Eighty four care homes were randomised: 39 to the GtACH programme and 45 to usual care. This imbalance was related to the stratification by site and type and uneven recruitment of homes by type across sites. Overall, 1657 residents consented and provided baseline measures. Consent was obtained for an average of 50% of residents from participating care homes, and an average of 19.5 participants resided in each care home. GtACH training was delivered to 1051/1480 staff in 146 group sessions, representing 71% of care home staff. Figure 1 shows the flow of care homes and residents through the study. Table 1 shows the characteristics of the care homes and residents.

**Table 1:** Baseline characteristics of care homes and residents assigned to Guide to Action for Care Homes (GtACH) or to usual care. Values are numbers (percentages) unless stated otherwise

Characteristics	Overall (n=84)	GtACH group (n=39)	Usual care group (n=45)
**Care homes**			
Type of care home:			
Nursing	11 (13)	5 (13)	6 (13)
Residential	34 (40)	16 (41)	18 (40)
Dual registration	39 (46)	18 (46)	21 (47)
Total No of care giving staff	3609	1491	2118
Median (IQR) No of care giving staff per home	33 (25-50)	38 (24-50)	47 (25-48)
Total No of beds	4112	1912	2200
Median (IQR) No of beds per home	41 (33-62)	43 (33-64)	41 (33-60)
Total No of residents	3561	1672	1889
Median (IQR) No of residents per home	36 (27-53)	39 (28-59)	34 (26-48)
Median (IQR) No of recruited residents per home	18 (15-22)	18 (12-22)	18 (15-22)
**Residents**			
Total No	1657	775	882
Mean (SD) age (years)	85.0 (9.3)	86.0 (8.6)	84.2 (9.7)
Men	532 (32.1)	231 (29.8)	301 (34.1)
Consent: resident	387 (23.4)	186 (24.0)	201 (22.8)
Consultee*	1270 (76.6)	589 (76.0)	681 (77.2)
Median (IQR) months in care home	18.6 (8.3-36.4)	18.8 (8.1-36.5)	18.1 (8.6-35.8)
Dementia	1109 (67.0)	506 (65.4)	603 (68.4)
Diabetes	320 (19.3)	150 (19.4)	170 (19.3)
Stroke	262 (15.8)	118 (15.2)	144 (16.3)
Coronary heart disease	234 (14.1)	100 (12.9)	134 (15.2)
Mean (SD) falls/person in 3 months before baseline	0.71 (1.82)	0.61 (1.57)	0.79 (2.02)
Mean (SD) PAM-RC score	8.61 (6.09)	8.57 (5.95)	8.66 (6.21)
Mean (SD) Barthel index score	8.57 (6.05)	8.86 (6.12)	8.30 (5.99)
EQ-5D-5L self-completion	0.49 (0.36)	0.52 (0.36)	0.46 (0.35)
EQ-5D-5L proxy	0.35 (0.37)	0.36 (0.37)	0.34 (0.36)
DEMQOL-U self-completion	0.82 (0.16)	0.83 (0.16)	0.81 (0.16)
DEMQOL-U proxy	0.74 (0.12)	0.74 (0.12)	0.74 (0.12)

* Care home managers or next of kin who helped to recruit residents.

PAM-RC=Physical Activity Measure-Residential Care Homes; EQ-5D-5L=five domain five level version of EuroQoL index (Crosswalk^18^ value set ranges from utility of 1 for best imaginable health to −0.59 for worst imaginable health); DEMQOL-U=Dementia Quality of Life utility measure.

### Outcomes

Data for the primary outcome, fall rate at 91-180 days after randomisation, were available for 630 residents assigned to GtACH and 712 assigned to usual care (table 2). The fall rate over this period was 6.0/1000 resident days in the GtACH group and 10.4/1000 resident days in the usual care group. The unadjusted incidence rate ratio of 0.57 (95% confidence interval 0.45 to 0.71, P<0.001) favoured GtACH; after adjusting for baseline falls rate, the incidence rate ratio was similar (0.63, 0.52 to 0.78, P<0.001). A significantly lower fall rate was found in the GtACH group at 1-90 days after randomisation, but not at 181-270 or 271-360 days after randomisation (table 2). No differences were found between groups in any of the other secondary outcomes; other than a lower proportion of residents sustaining fractures in the GtACH group at 181-360 days after randomisation (table 3 and table 4).

**Table 2:** Primary and secondary fall rate outcomes in care home residents assigned to Guide to Action for Care Homes (GtACH) or to usual care

Fall rate outcome	GtACH group				Usual care group			Minimally adjusted* incidence rate ratio (95% CI)	P value	Fully adjusted† incidence rate ratio (95% CI)	P value
	No at risk	Mean (SD) falls per participant	Mean (SD) fall rate per 1000 resident days		No at risk	Mean (SD) falls per participant	Mean (SD) fall rate per 1000 resident days				
**Primary outcome**											
91-180 days	630	0.49 (1.13)	6.04 (14.02)		712	0.89 (2.60)	10.38 (29.52)	0.57 (0.45 to 0.71)	<0.001	0.63 (0.52 to 0.78)	<0.001
**Secondary outcomes**											
1-90 days	708	0.55 (1.36)	6.93 (20.56)		826	0.88 (2.37)	10.24 (27.26)	0.6 (0.49 to 0.73)	<0.001	0.74 (0.60 to 0.92)	0.006
181-270 days	547	0.60 (1.29)	7.28 (16.67)		633	0.73 (1.85)	9.21 (28.77)	0.85 (0.69 to 1.05)	0.13	0.91 (0.74 to 1.12)	0.37
271-360 days	502	0.55 (1.14)	6.22 (12.88)		573	0.79 (2.37)	9.22 (27.36)	0.79 (0.60, to 1.03)	0.08	0.93 (0.71 to 1.22)	0.61

* Adjusted for design factors only: care home type and site as fixed effects and care home as random effect.

† Adjusted for care home type, site, and baseline falls rate as fixed effects and care home as random effect.

**Table 3:** Secondary outcomes comprising scores in care home residents assigned to Guide to Action for Care Homes (GtACH) or to usual care

Days	GtACH group			Usual care group			Minimally adjusted* mean difference (95% CI)	P value	Fully adjusted† mean difference (95% CI)	P value
	No	Mean (SD)		No	Mean (SD)					
Barthel index score:										
90	643	8.24 (6.12)		726	7.87 (5.94)		0.08 (−0.96 to 1.13)	0.87	−0.03 (−0.69 to 0.64)	0.94
180	584	8.12 (6.05)		648	7.54 (5.86)		0.16 (−0.89 to 1.20)	0.77	−0.02 (−0.48 to 0.43)	0.93
270	514	8.52 (6.17)		576	7.18 (5.98)		0.90 (−0.29 to 2.10)	0.14	0.46 (−0.10 to 1.01)	0.11
360	447	8.11 (6.20)		519	6.86 (5.92)		0.82 (−0.32 to 1.96)	0.16	0.44 (−0.26 to 1.15)	0.21
PAM-RC scores:										
90	652	7.99 (6.01)		736	8.16 (5.98)		−0.41 (−1.51 to 0.69)	0.47	−0.1 (−0.55 to 0.35)	0.66
180	578	8.11 (6.05)		633	7.74 (6.08)		0.07 (−1.04 to 1.17)	0.91	0.23 (−0.28 to 0.75)	0.38
270	491	8.13 (5.98)		576	7.59 (6.12)		0.32 (−0.90 to 1.54)	0.61	0.43 (−0.24 to 1.10)	0.21
360	439	7.96 (5.63)		520	7.19 (6.03)		0.45 (−0.57 to 1.47)	0.39	0.49 (−0.16 to 1.14)	0.14

PAM-RC=Physical Activity Measure-Residential Care Homes.

* Adjusted for design factors only: care home type and site as fixed effects and care home as random effect.

† Adjusted for care home type, site, and baseline falls rate as fixed effects and care home as random effect.

**Table 4:** Secondary outcomes comprising event rates in care home residents assigned to Guide to Action for Care Homes (GtACH) or to usual care

Events by follow-up (days)	GtACH group		Usual care group		Minimally adjusted* odds ratio (95% CI)	P value	Fully adjusted† odds ratio (95% CI)	P value
	No	No (%) with adverse outcome	No	No (%) with adverse outcome				
Fractures (≥1):								
0-180	775	33 (4.3)	822	42 (4.8)	1.19 (0.70 to 2.01)	0.53	–	–
181-360	600	9 (1.5)	685	26 (3.8)	0.34 (0.15 to 0.75)	0.007	–	–
Deaths:								
1-360	775	233 (30.1)	882	281 (31.9)	0.93 (0.73 to 1.20)	0.58	–	–
Fallers ((≥1):								
1-90	708	194 (27.4)	826	266 (32.2)	0.70 (0.50 to 1.00)	0.05	0.75 (0.53 to 1.05)	0.09
91-180	630	167 (26.5)	712	216 (30.3)	0.76 (0.56 to 1.03)	0.08	0.81 (0.60 to 1.10)	0.18
181-270	547	165 (30.2)	633	187 (29.5)	1.00 (0.73 to 1.37)	0.99	1.06 (0.78 to 1.45)	0.70
271-360	502	147 (29.3)	573	175 (30.5)	0.88 (0.60 to 1.29)	0.52	0.94 (0.65 to 1.37)	0.75

* Adjusted for design factors only: care home type and site as fixed effects and care home as a random effect.

† Adjusted for care home type, site and baseline value of the outcome as fixed effects and care home as a random effect.

### Health economic outcomes

The final dataset for the economic analysis comprised 1603 residents (732 in the GtACH group and 871 in the usual care group). Completion rates for data were high, with no more than 283/1603 (17.7%) items missing from any individual variable (DEMQOL based QALYs) and complete datasets available for 1260/1603 (78.6%) residents. Six of the 1603 residents (0.4%) were missing cost data, 13 (0.8%) and 15 (0.9%) were missing baseline EQ5D and DEMQOL utility data, and 262 (16.3%) and 283 (17.7%) were missing EQ-5D and DEMQOL based QALYs, respectively.

Table 5 shows the incremental costs per resident for the GtACH programme and the difference in outcomes between the groups (QALYs and falls over 12 months). Table 6 shows the incremental cost effectiveness ratios. The primary analysis showed the incremental cost per EQ-5D-5L-P based QALY to be £4544 and per DEMQOL-P-U based QALY to be £20 889. The cost per fall averted was £191. The results of the sensitivity analysis were similar to those of the base case. The cost effectiveness acceptability curve showed a 92% probability that the GtACH programme was cost effective at a £20 000 per QALY threshold using QALYs based on the EQ-5D-5L-P, and a 57% probability using QALYs based on the DEMQOL-P-U. Supplementary table S3 shows the costs to deliver the GtACH programme per resident. Supplementary table S4 shows the incremental cost for each individual cost component. Supplementary table S5 shows mean utility scores by follow-up period and QALYs for DEMQOL-P-U and EQ-5D-5L at 12 months. Supplementary figures S6-S8 show scatterplots and cost effectiveness acceptability curves for EQ-5D-5L-P based QALYs, DEMQOL based QALYs, and the cost per fall averted, respectively.

**Table 5:** Health economics analyses for care home residents assigned to Guide to Action for Care Homes (GtACH) and to usual care groups

	GtACH group			Usual care group			Incremental mean (95% CI)	
	No	Mean (SD) cost (£)		No	Mean (SD) cost (£)		Primary analysis	Multiple imputation analysis
**Costs**								
Base case*	732	3955.29 (3949.38)		865	3935.54 (3879.9)		108.26 (−271.06 to 487.58)	108.26 (−232.89 to 449.41)
**Sensitivity analyses**								
Repeat GtACH use†	732	3978.2 (3955.87)		865	3935.54 (3879.9)		131.81 (−247.77 to 511.4)	131.81 (−209.28 to 472.9)
Extra mortality costs‡	732	4103.96 (4121.02)		865	4047.89 (3989.66)		124.98 (−268.68 to 518.64)	124.98 (−230.84 to 480.8)
Repeat GtACH use and extra mortality costs§	732	4126.87 (4127.1)		865	4047.89 (3989.66)		148.52 (−245.4 to 542.45)	148.52 (−207.33 to 504.38)
**Outcomes**								
EQ-5D-5L Proxy based QALYs	622	0.266 (0.317)		718	0.232 (0.291)		0.024 (0.004 to 0.044)¶	0.023 (0.003 to 0.043)¶
DEMQOL-P-U based QALYs	611	0.578 (0.24)		708	0.581 (0.235)		0.005 (−0.019 to 0.03)¶	0.005 (−0.018 to 0.029)¶
Falls over 12 months	732	1.889 (3.662)		871	2.747 (7.414)		−0.568 (−0.97 to −0.166)¶	−0.574 (−0.961 to −0.186)¶

£1.00 (€1.19; $1.35).

EQ-5D-5L=five domain five level version of the EuroQoL index (Crosswalk^18^ value set ranges from utility of 1 for best imaginable health to −0.59 for worst imaginable health); DEMQOL-U=Dementia Quality of Life utility measure (utility ranges from 0.363 to 0.937 for DEMQOL-U-P (proxy) and 0.986 to 0.243 on DEMQOL-U, both value sets derived using time trade-off methods).

* Based on intervention costs assuming GtACH tool is used once per resident as this was reflective of what the process evaluation team observed. The base case analysis also excluded secondary care costs related to end of life care and mortality.

† This analysis costs the intervention assuming the maximum number of repeat GtACH’s each care home could provide would be one per month if the resident fell.

‡ This analysis incorporates secondary care costs related to mortality.

§ This sensitivity analysis incorporates both repeated GtACH use and mortality costs.

¶ Mean difference (95% confidence interval).

**Table 6:** Economic analyses of the Guide to Action for Care Homes (GtACH) programme

Incremental cost effectiveness ratios	Primary analysis (£)	Multiple imputation analysis (£)
**Base case**		
Per EQ-5D-5L-P based QALY	4543.69	4651.63
Per DEMQOL-P-U based QALY	20 889.42	20 557.80
**Sensitivity analyses**		
With repeat GtACH use:		
Per EQ-5D-5L-P based QALY	5532.14	5663.56
Per DEMQOL-P-U based QALY	25 433.80	25 030.04
With extra mortality cost:		
Per EQ-5D-5L-P based QALY	5245.37	5369.98
Per DEMQOL-P-U based QALY	24 115.39	23 732.56
With repeat GtACH and extra mortality cost:		
Per EQ-5D-5L-P based QALY	6233.50	6381.58
Per DEMQOL-P-U based QALY	28 658.26	28 203.32
**Incremental cost per fall averted**		
Base case	190.62	188.72
With repeat GtACH use	232.09	229.77
With extra mortality cost	220.06	217.86
With repeat GtACH use and extra mortality cost	261.52	258.91

£1.00 (€1.19; $1.35).

EQ-5D-5L=EuroQoL index; DEMQOL-P-U=Dementia Quality of Life utility measure.

## Discussion

The Guide to Action for Care Homes (GtACH) programme was associated with a statistically significant reduction in fall rate in care home residents in the period 3-6 months after randomisation, which was the primary outcome of this study. The reduction in falls was achieved with no effect on death, dependency, or activity.

The GtACH programme was within conventional thresholds of cost effectiveness when health related quality of life was estimated using the EQ-5D-5L-P.

Considering secondary outcomes, these aligned with the primary outcome, showing a significant reduction in falls at months 1-3 after randomisation. There was also an indication that use of the GtACH could be associated with fewer fractures—this has potentially important cost saving implications for health services.

### Strengths and weaknesses of this study

The strengths of this study relate to the large and representative sample size, the measures taken to avoid contamination, and the comprehensive approach to costing and health economics. Few data were missing, partly because data were collected quarterly. We consider that training 71% of care home staff was a success, given that such staff often work part time or out of office hours, and given the extent of staff turnover in the care home sector.

A weakness of the study was that we were unable (owing to ethical approvals) to collect falls data from residents not in our study who may also have been exposed to the intervention, or not, depending on the arm to which they were randomised. Our recruitment process might have selected care homes with staff who had a particular interest in falls prevention, meaning that usual care in our control homes might have been better than usual care seen more widely—this might have been expected to reduce the size of the treatment effect and is unlikely to negate our findings. It is possible that the intervention could have influenced the reporting of falls differentially between intervention and control arms because of the nature of GtACH. Although it is difficult to be sure about the directionality of such a difference, the focus on reporting falls to trigger the GtACH intervention could well have increased falls reporting in intervention homes compared with control homes. Thus it is unlikely that such a difference would have contributed to an overestimation of treatment effect. The loss of the treatment effect after six months could be due to a loss of statistical power owing to high attrition from death, but even if this was a genuine loss of effect, the short life expectancy of care home residents might make a short lived treatment effect acceptable. Another explanation for the loss of treatment effect after six months could be a waning of falls awareness, and it is possible that further support or training at this time could have perpetuated the treatment effect. This study was not powered to detect a difference in fractures between groups. We consider that the significantly lower proportion of participants in the GtACH group who sustained one or more fractures in the period 181-360 days after randomisation is interesting, but the analysis was based on small numbers and there was no corresponding reduction in falls over that period.

The number of care homes between groups showed a slight imbalance (39 in the GtACH arm and 45 in the control arm) because we had a limited number of care homes with some of the characteristics required for stratified randomisation. Despite this, the randomisation list still had a 1:1 ratio in each stratum and we therefore do not think that this influenced our findings. The median number of staff per home differed between the control (n=47) and GtACH (n=38) arms, despite a similar median number of beds. This means that the staffing ratios were lower in the intervention homes. Staffing of care homes is complex and is influenced by factors such as funding models; the geographical location of care homes, which impacts on availability of staff; and the ethos and culture of the provider organisation.^26^ Staffing ratios in care homes are not closely related to resident dependency in the UK as they are in other countries. We did not find a substantive difference in other baseline variables that would indicate these staffing ratios reflect differing resident dependency across the intervention and control arms.

A limitation was the number of homes that could not participate because they did not agree to appoint a falls champion or stopped communicating with the researcher. Thus, the study might have selected care homes that were better led, organised, or resourced. It is well recognised that care homes that support research are by definition of volunteering to participate in research, always atypical.^27^ We can, however, be confident that the age, functional dependency, sex, and comorbidities of the residents were similar to those of other care home research^3^ and that our protocol enabled us to sample from a wide range of organisations for size, registration status, and specialisation. These findings are likely to be as generalisable as any care home research. Our next project is to consider how we can implement these findings across the wider range of care homes that do not routinely participate in research studies.

### Strengths and weaknesses in relation to other studies

Cochrane systematic reviews have shown that the fall rate in older people living in their own home can be reduced, but research presented in the 2018 Cochrane review of 13 care facilities were inconclusive about multifactorial interventions for falls prevention.^6^ The studies were classified as very low or low quality. Our study provides different findings.

An important question is why this study might have been successful in showing a reduction in falls, whereas others have not found such outcomes. An important difference between this study and previously published work relates to the earlier processes of co-design, piloting, and feasibility trials,^7^ ^8^ which enabled us to design an intervention that was sensitive in the context of care homes and cognisant of the specific challenges of falls prevention in this sector. This is in keeping with an increasing body of evidence, which found that interventions that assess and take account of the care home context,^28^ and which empower care home staff and organisations as partners in design and implementation,^29^ are more likely to be successful.

A limitation of our study common to falls studies was that although the outcomes were collected by researchers blinded to allocation, participants and care home staff were not blinded and they recorded the falls in care records. The direction of any bias resulting from this is unclear and it is likely that any bias would be small because UK care homes have a statutory duty of care to record falls. We checked the falls recording processes at each follow-up, and all care homes used the same falls definition. We consider that our interest in falls might have increased the accuracy of falls recording in the care homes, as the care home staff recognised they were participating in a study.

Although GtACH was found to be cost effective using the EQ-5D-5L, it was of borderline cost effectiveness when the DEMQOL-P-U was used. Research published after our study had started indicates that the EQ-5D performs better than the DEMQOL in care homes.^21^ ^30^ EQ-5D has been shown to be more responsive to change,^31^ which is the justification for our conclusion that the GtACH programme was cost effective. The DEMQOL focuses more on the emotional impact of dementia, whereas in this study the outcome was not dementia specific, and not all residents had dementia. The mean utility for self-reported quality of life indices was higher than that for proxy report. This might reflect that those with earlier dementia, and hence able to complete self-report questionnaires, had better quality of life. It might also be a consequence of the limited agreement between proxy and self-report quality of life indices when used in the care home population,^32^ which is well described as a limitation that affects all studies conducted in care homes and is an area of ongoing research.

### Policy implications

This study provides findings that confirm the hypothesis that an intervention which includes all of awareness raising, education, screening, decision support, and implementation support can reduce falls in care homes. It is possible that the intervention succeeded because of its comprehensiveness and as a result of the recognition it gave to the pivotal role played by care home staff in designing, implementing, and delivering the GtACH programme in this setting.^33^ Clinicians and policy makers should, when working to prevent falls in care homes, implement interventions that are similarly comprehensive in scope and that include each of the components included in GtACH. There are always caveats associated with extrapolating such findings to care homes that might differ structurally, culturally, or organisationally from those included in the study, but we do not believe that these issues of generalisability can be addressed by further trials. Rather, it is implementation research that is required to explain how to realise these benefits across a wider range of settings.

This was a complex intervention, and key to understanding the implications for service delivery is that a process evaluation was completed concurrently to this trial and provided insights into how care homes received and delivered GtACH, including how behaviours and processes changed in relation to the programme. We have captured these data—both quantitative and qualitative—in the process evaluation and plan to publish the results separately to enable all relevant detail to be shared.

Although this work was conducted in the UK, where care homes are run by private providers and not managed by the NHS, these findings are likely to be generalisable to other long term care settings for older people internationally, where the incidence of falls is high and where nurses and care assistants without specialist training in falls prevention predominantly provide day-to-day delivery of care.^34^ ^35^ In the same way that further research is required and planned, to understand the generalisability of our findings to other non-participant UK care homes, similar work is required to explore how to accommodate our findings in long term sectors internationally where conditions might differ from those in the UK.

### Unanswered questions and future research

Future work should aim to implement the GtACH programme in care homes where a systematic and equivalent fall prevention programme is not in place. Implementation in care homes is not straightforward and must take account of substantial variation in ethos and organisational structure between care home operators.^28^ It will be important, therefore, to research how to implement GtACH consistently and sustainably across different health and social care systems, and at scale. We are currently undertaking further research to understand how to consistently implement the GtACH programme in care homes. The research will also develop digital materials and online training to support this.

### Conclusion

Our multifactorial falls prevention intervention in UK care homes was associated with a reduction in fall rate and cost effectiveness, without a reduction in activity or increase in dependency. Further research working closely with the care home sector is required to understand how to implement this type of intervention consistently across the full range of care home provider organisations.

**What is already known on this topic**

Falls are common in older residents of care homes and are associated with high risk of injury, admissions to hospital, and important cost to healthcare systems
Although interventions for falls prevention have been shown to be effective in other settings, previous systematic reviews suggested that the benefits were uncertain in care home residents
Interventions in care homes tend to be more effective if they are co-designed with residents and staff and take account of the care home context

**What this study adds**

A multifactorial falls prevention programme in care homes for older people, co-designed with care home staff and residents, that involved awareness raising, education, screening, decision support, and implementation support was associated with a reduction in falls rate
The intervention was found to be cost effective
No adverse effects of the intervention were found on residents’ activity levels or physical dependency

## Data Availability

Data requests should be submitted to the corresponding author for consideration. Data collected for the study, including anonymised individual participant data and a data dictionary defining each field in the set, will be made available to others after review.

## Web extra

Extra material supplied by authors

## Notes

Contributors: PAL (chief investigator) designed and led trial delivery and wrote the paper. She is the guarantor. JCH and ES led trial delivery and contributed intellectual content to the paper. FA, JD, KM, and KR collected and analysed data and wrote the paper. SA, AC, and SS did the statistical analysis, and accessed and verified the data. TS, ECFW, and LI did the health economic analysis. SC, CF, GM, ALG, JRFG, and KR led data collection, and contributed to data analysis and writing the paper. MG was the patient and public involvement representative and contributed to all aspects of the study. The corresponding author attests that all listed authors meet authorship criteria and that no others meeting the criteria have been omitted.

The lead author (PAL) affirms that the manuscript is an honest, accurate, and transparent account of the study being reported; that no important aspects of the study have been omitted; and that any discrepancies from the study as planned (and, if relevant, registered) have been explained

Dissemination to participants and related patient and public communities: We have presented results at national and international conferences and in addition have been awarded an implementation grant from the National Institute for Health Research Applied Research Collaboration to investigate how to improve uptake. We anticipate that further, lay facing, publications and resources will be developed as part of this further work. The findings will also be incorporated into national guidelines.

Provenance and peer review: Not commissioned; externally peer reviewed.
