# Supplementary material for: Multifactorial falls prevention programme compared with usual care in UK care homes for older people: multicentre cluster randomised controlled trial with economic evaluation
Source: BMJ. 2021 Dec 7;375:e066991. doi: 10.1136/bmj-2021-066991 (PMC8649897; doi:10.1136/bmj-2021-066991)

*Supplementary Material S1: Intervention Description [TIDieR Checklist]*

The GtACH Fall Prevention Programme is summarised using the template for intervention description and replication (TIDieR) checklist (1) detailed below:

**Item 1:** Name

The Guide to Action Care Homes (GtACH) Falls Prevention Programme.

**Item 2:** Rationale

The relatively untrained nature of care home staff, the complex nature of falls risk factors in care residents and the need for multiple interventions to address multiple risk factors requires a systematic home-wide programme including staff education and support, in the use of risk assessment and decision support tools.

**Item 3:** Materials

- GtACH training slides – these were used by the NHS Falls Lead to train each intervention home
- The GtACH manual – given to care home staff to support implementation of the GtACH paper screening and assessment tool. It included a master copy of the paper tool, information about the study, a copy of the training session slides, falls information (including definition of a fall, why falls are important and causes of falls), instructions on how to complete the GtACH paper screening and assessment tool, a Falls Incident Analysis template, a Medication and Falls Chart and information on how to obtain further expert advice or support from the local falls' expert.
- The GtACH screening and assessment tool comprised 33 items related to falls risk factors grouped into four domains: falls history, medical history, movement/environment, and personal needs. The presence of risk factors prompts up to 30 individual staff actions.
- Attendance certificate – given to care home staff at the end of training
- A4 sized poster – given to care homes for display in the home to act as a reminder to implement the GtACH programme in the home.

**Item 4:** Procedures

- Care home staff training provided by the local NHS Falls Lead, lasted one hour per session and included: purpose of the study, purpose of the training, prevalence of falls in care homes, GtACH history, and how to complete and where to file completed forms. It emphasised consistent delivery, referenced the materials listed above especially the GtACH manual. Case studies and role play were used.

**Item 5:** Intervention provider

- The NHS Falls Lead was a registered nurse, physiotherapist or occupational therapist who was trained and specialised in falls prevention and bone health.

- A care home Falls Champion, with roles for awareness raising and liaison between staff and the NHS Falls Lead, was identified in each care home. No additional training was provided.
- Trained staff were taught to complete the GtACH screening and assessment tool with every resident, within four weeks after training, in private, and discussed with family, friends and other care home staff. Completed GtACH documentation was to be placed in the resident's care records and expected to contribute to the care plan for each resident. Re-assessment was expected if the resident developed a new health condition, or fall, or every three-six months.

**Item 6:** Mode of delivery

- Training was given face to face.

**Item 7:** Where the training occurred

- Training was provided in a room within the care facility which was deemed appropriate for training by the care facility manager/owner.

**Item 8:** Duration/Intensity

- Each session was one hour. As it was not feasible for all care home staff to attend a single training session, repeated training sessions were offered according to care home staff availability. Staff were not remunerated, their roles were not back filled, repeated sessions maximized opportunity for all staff to attend.

**Item 9:** Tailoring

- Completed GtACH documentation was to be placed in the resident's care records and expected to contribute to the care plan for each resident. Re-assessment was expected if the resident developed a new health condition, or fall, or every three-six months.

**Item 10:** Modifications

- The training period for care homes was extended from two weeks to four weeks (post randomisation) to enable fall leads time to train very large homes (+50 staff members).

**Item 11/12 :** Fidelity

- Fidelity of the intervention is reported separately in the process evaluation which was conducted in parallel to the trial.

*Supplementary material S2 – Statistical Analysis Plan*

## Section 1: Introduction

### Background and rationale:

Preventing falls and injuries in those over 65 years of age is a public health priority (RoSPA 2013) and The King's Fund recommends structured patient-centred care in care home settings (Naylor 2013). The recently published NICE Quality Standard 86, 'Falls in older people: assessment after a fall and preventing further falls' (NICE, 2015), recommends that all health and social care practitioners involved in assessing, caring for and treating older people who experience a fall should have sufficient and appropriate training and competencies to deliver the actions and interventions.

Community fall prevention interventions reduce falls by about 30%, but literature to date has found no conclusive reduction in falls in care homes (Gillespie 2012, Cameron 2012). To decrease fall rates it has been suggested that interventions need to be targeted at high risk groups such as elderly care home populations and include specific components (Close, 2005). They need to be delivered by the whole team (Bouwen 2008, Jensen 2004) to the whole environment.

The GtACH intervention aims to reduce fall rates in care homes by facilitating change in practice of care home staff. It was co-produced by a group of care home staff, clinicians, researchers, public, voluntary and social care organisations and includes care home staff training, support and documentation.

### Objectives

The primary objective of the FinCH trial is to compare the rate of falls per participant in the 2 trial arms (GtACH arm and usual practice control arm) during the 3 month period comprising 4,5 and 6 months post randomisation.

Secondary objectives listed in the protocol are as follows::

i. Comparison of fall rates between the two groups during the 3-month period comprising 7, 8 and 9 months post randomisation.
ii. Comparison of fall rates between the two groups during the 3-month period comprising 10, 11 and 12 months post randomisation.
iii. Comparison of frequency of falls injuries between the two groups for falls occurring between baseline and 6 months, and between 7 and 12 months post randomisation.
iv. Comparison, between the two groups, of frequency and type of fractures occurring between baseline and 6 months, and between 7 and 12 months post randomisation.
v. Comparison, between the two groups, of physical activity , measured using PAM-RC, at 6 months, and 12 months post randomisation.
vi. Comparison, between the two groups of functional ability, measured using Barthel index, at 6 months, and 12 months post randomisation.
vii. Comparison, between the two groups, of quality of life, measured using DEMQOL and EQ5-D at 6 months, and 12 months post randomisation.
viii. Comparison, between the two groups, of medication use between baseline and 6 months, and between 7 and 12 months post randomisation.
ix. Comparison, between the two groups, of number of days in hospital between baseline and 6 months, and between 7 and 12 months post randomisation.
x. Comparison, between the two groups, of percentage of deaths

**Changes from the protocol**

The following objective has been added, on the recommendation of the DMC

i Comparison, between the two groups, of percentage of residents falling, between 3 and 6 months post-randomisation, and between 7 and 12 months post-randomisation

Changes to original secondary objectives

i original objective viii (Comparison, between the two groups, of medication use between baseline and 6 months, and between 7 and 12 months post randomisation) has been amended to Comparison, of risk of falling, among those on 4 or more medications at baseline

Full details of the trial are given in the protocol.

**Section 3: Study Methods**

**Trial Design**

FinCH is a cluster randomised controlled, 2 arm, parallel group trial comparing the GtACH fall prevention intervention against usual care for people living in care homes in England (with and without nursing). Care home is the unit of randomisation.

**Randomisation**

Care homes are randomised on a 1:1 basis to one of two parallel arms: intervention (GtACH fall prevention programme) or control (usual care).

Randomisation is based on a bespoke computer generated pseudo-random code using variable block randomisation (block sizes 2 and 4) within strata (site [Lincolnshire, Derby, Northumbria, Leicester, Stafford, Norwich, Nottingham City, Nottinghamshire, Bradford and Solent] and care home type [nursing/residential/dual registration]) provided by the Norwich CTU via a secure web based randomisation service.

**Sample size**

The most recent sample size calculation was based on a falls rate of 2.5 falls per person per year (0.625 falls in 3 months) with 80% power and a two-sided significance level of 5%. Based on an average cluster size of 19 (SD 9.5) residents per cluster, a coefficient of variation (CV) of 0.5 and allowing for 16% attrition, the sample size target was 78 care homes - 1482 residents in total. Full details of this sample size calculation, and the original calculation, may be found in sections 9.1.1, 9.1.2 and 9.1.3 of the FinCH protocol.

**Framework**

The FinCH trial is to determine whether fall rates are reduced following the implementation of the GtACH intervention and therefore is testing for superiority. Secondary outcomes will also be tested for superiority.

**Statistical interim analyses and stopping guidance**

There are no interim analyses planned for FinCH.

**Timing of final analysis**

All analyses will take place at one time point once all data are cleaned and locked.

**Timing of outcome assessments**

Data on falls, medication use, time in hospital and use of primary care and community services for the previous 3 months ("baseline") are collected at the time of resident recruitment, then at 3 months following randomisation, and at 3 monthly intervals thereafter, with the final collection of these items taking place at approximately 12 months post-randomisation. Data on Physical activity (PAM-RC), Activities of Daily Living (Barthel), Health related Quality of Life in Dementia (DEMQOL-U-5D / DEMQOL-P-4D, EQ-5D-5L/EQ-5D-5L proxy) will be collected from participating residents at the same 3 month intervals. Frequency and type of fractures occurring during the period of baseline and outcome data collection will obtained from Hospital Episode Statistics at the end of the 12 month follow-up. Full details of timing of collection of outcome measures are included in the FinCH trial protocol.

**Section 4: Statistical Principles**

**Confidence intervals and p-values**
All statistical tests will be 2-sided and performed using a 5% significance level. All confidence intervals presented will be 95% and 2-sided. No adjustment for multiplicity is planned.

**Adherence and protocol deviation**
Compliance with the intervention is based on the percentage of care giving staff in each care home trained to use the GtACH tool. It is calculated as follows:

$$\% \text{ compliance} = \frac{number\ of\ care\ giving\ GtACH\ trained\ staff\ at\ care\ home}{number\ of\ care\ giving\ staff\ at\ care\ home} * 100\%$$

Percentage compliance will be calculated and presented for each home in the intervention arm; average compliance for all intervention care homes will also be presented.

**Analysis populations:**
Analyses will be undertaken on an Intention to Treat basis in which care homes (and corresponding participating residents) will be analysed in the group to which they were allocated regardless of their compliance with the intervention). Those who died between care home randomisation and the three month follow-up data collection will be regarded as having been exposed to the intervention (GtACH/control), recorded as lost to follow-up at the time of death, and included in the consort diagram. The consort diagram will include the number of people who were randomised in error – eg those who were randomised but did not fulfil all the eligibility criteria.

**Section 5: Trial Population**

**Screening data**
The following summaries will be presented for all screened care homes, both overall and by study site:

Number of screened care homes
The number of care homes not recruited for the following reasons: would not benefit the care home; have not got the time; not interested in research; other

**Eligibility**

Care homes: the number of ineligible care homes will be reported, along with reason(s) for ineligibility
Residents: the number of eligible and ineligible residents will be reported, along with reason(s) for ineligibility
A list of inclusion and exclusion criteria for care homes and residents is provided in section 7.5.2 of the protocol.

**Recruitmen**t
The Consort diagram will be used to summarise the following information:

Number of Care homes assessed for eligibility
- Number of care homes eligible at screening
- Number of care homes ineligible at screening

Number of care homes recruited
Number of residents recruited
Number of care homes randomised to each trial arm

Number of care homes lost to follow-up
Number of participating residents lost follow-up
Number of care homes discontinuing the intervention
Number of residents included in the primary analysis

**Withdrawal/follow up**
Reason for, and level and timing of, withdrawal of consent of care homes and participating residents will be indicated in the Consort diagram

**Baseline characteristics**

Baseline characteristics of randomised care homes and participating residents will be presented according to Table 1 and Table 2 respectively, both overall and by randomised group. No formal hypothesis tests will be undertaken. Categorical data will be summarised by numbers and percentages; continuous data will be summarised by mean, SD and range if data are normal, and median, IQR and range if data are skewed.

Table 1: Baseline characteristics of randomised care homes

| **Baseline characteristics of randomised homes by allocated group** | Overall (n=) | Group A (n=) | Group B (n=) |
|---|---|---|---|
| Number of care homes by site | | | |
| - Lincolnshire | | | |
| - Derby | | | |
| - Northumbria | | | |
| - Leicester | | | |
| - Stafford | | | |
| - Norwich | | | |
| - Nottingham City | | | |
| - Nottinghamshire | | | |
| - Bradford | | | |
| - Solent | | | |

| | | | |
|---|---|---|---|
| Number of care homes by type | | | |
| - Nursing | | | |
| - Residential | | | |
| - Dual Registration | | | |
| Number of care homes by ownership | | | |
| - charity | | | |
| - private | | | |
| Total number of care giving staff<br>Mean (SD) care giving staff per home | | | |
| Total number of beds<br>Mean (SD) beds per home | | | |
| Total number of residents<br>Mean (SD) residents per home | | | |
| Percentage of residents recruited out of those eligible | | | |
| Percentage of residents recruited out of those resident | | | |

Table 2: Baseline characteristics of participating residents

| **Baseline characteristics of participating residents by allocated group of care home** | Overall | Group A | Group B |
|---|---|---|---|
| Age at consent to FinCH (years): mean (SD) | | | |
| Male: N (%) | | | |
| Consent: N(%) | | | |
| - Resident | | | |
| - Consultee | | | |
| Time in care home (months ): mean (SD) | | | |
| Active medical diagnoses: N(%) | | | |
| - Dementia | | | |
| - Diabetes | | | |
| - Stroke | | | |
| - CHD | | | |
| Number of falls during period 3 months prior to baseline data collection | | | |
| Number of Medications in period 3 months prior to baseline data collection<br>- None<br>- One to three medications<br>- Four or more medications | | | |
| Physical activity (PAM-RC) score at baseline: mean(SD) | | | |
| Activities of Daily Living (Barthel) score at baseline: mean(SD) | | | |
| DEMQOL-U-5D at baseline | | | |
| DEMQOL-P-4D at baseline | | | |
| EQ-5D-5L at baseline | | | |
| EQ-5D-5L proxy at baseline | | | |
| | | | |

**Section 6: Analysis**
**Outcome definitions**

**i) Falls rates:**
**Primary outcome**
The primary outcome is the rate of falls per participating resident during the 3 month period comprising months 4, 5 and 6 post-randomisation. For the purpose of the analyses, a month will be taken to consist of 30 days. Baseline, or time zero, for each care home will be the date of randomisation of that care home. For participating residents at each care home, month 4 will therefore start 91 days after randomisation, and month 6 will finish 180 days after randomisation. Therefore for each participating resident the numerator of the falls rate will be the number of falls occurring in the care home between 91 and 180 days. The denominator will be 90 days, for those participating residents who have lived at the care home for the entire 90 day period; for those participating residents who have spent time in hospital or elsewhere during the 90 day period, the denominator will be the number of days during that 90 day period spent as resident in the care home. The falls rate will be expressed as the number of falls per 1000 participating resident days for each group. For individuals who stopped participating in the trial before 180 days, data will be included up until the date of withdrawal.

**Secondary outcomes**
Rate of falls occurring during the 3 month period comprising months 7, 8 and 9 post-randomisation, and the 3 month period comprising months 10, 11 and 12 post-randomisation. As above, a month will be assumed to consist of 30 days. The 3 month period comprising months 7, 8 and 9 will therefore commence on day 181 and end on day 270. Likewise the 3 month period comprising months 10, 11 and 12 will commence on day 271 and end on day 360. Falls rates will be calculated as described for the primary outcome, and expressed as the number of falls per 1000 participating resident days for each group.

**ii) Other secondary outcomes:**
The number and percentage of residents having one or more falls, between 3 and 6 months post-randomisation, and between 7 and 12 months post-randomisation.

The number and percentage of residents having no medications at baseline, who have one or more falls, between 3 and 6 months post-randomisation, and between 7 and 12 months post-randomisation.

The number and percentage of residents having 4 or more medications at baseline, who have one or more falls, between 3 and 6 months post-randomisation, and between 7 and 12 months post-randomisation.

Fractures (from HSCIC data) occurring between baseline and 6 months and between 7 months and 12 months:

- Number of fractures of any type, per resident
- Number of residents having one or more fractures of any type
- Number of hip fractures, per resident
- Number of residents having one or more hip fracture
- Number of wrist fractures, per resident

- Number of residents having one or more wrist fracture

Physical activity (measured by the PAM-RC questionnaire) at 3, 6, 9 and 12 months following randomisation. The PAM-RC questionnaire comprises 5 questions addressing ability (mobility and balance), and activity (walking frequency, wandering and outdoor mobility). Each question has graded responses, scored between 0-3 and 0-6. The total PAM-RC will be calculated by summing the scores for each question, giving a range of scores between 0 and 21, with 0 indicating no activity, and 21 indicating that the resident is fully mobile in all aspects of the questionnaire.

Activities of daily assessment (measured by the Barthel Index) are recorded at baseline, 3, 6, 9 and 12 months following randomisation. The Barthel Index of Activities of Daily Living comprises ten questions concerning resident's ability to self-care. Each item is scored between 0 and 1, 2 or 3, with minimum possible score 0 and maximum possible score 20; lower values indicating less independence with respect to Activities of Daily Living. Total ADL scores will be treated as continuous data.

Quality of life in dementia, measured using DEMQOL-U for residents with capacity and DEMQOL-Proxy for residents lacking capacity, is recorded at baseline, and at 3, 6, 9 and 12 months following randomisation. DEMQOL comprises 28 questions and DEMQOL-Proxy 31 questions, each scored between 1 and 4. Scores are summed to give total DEMQOL and DEMQOL-Proxy scores with possible ranges 28 to 112 and 31 to 124 respectively; higher scores imply better Quality of Life. DEMQOL and DEMQOL-Proxy also each include a single global health question – this is not included in the total DEMQOL or DEMQOL-Proxy score. Total DEMQOL and DEMQOL-Proxy scores will be analysed as continuous data.

Health status measured using EQ-5D-5L (where the participating resident has capacity), and EQ-5D-5L proxy (where the participating resident does not have capacity), is recorded at baseline and at 3, 6, 9 and 12 months post-randomisation. At each timepoint responses to the 5 component questions of the EQ-5D_5L/EQ5D-5L proxy will be converted to a weight. The values of weights range from -0.285 to 0.950, with higher weights indicating more favourable health states. A further question asks residents / proxy respondents to score how well or poorly they rate their health on that particular, on a scale ranging from 0 (worst imaginable health state) to 100 (best imaginable health state).

The number of days spent by residents in hospital (from HSCIC data) between baseline and 6 months, and between 7 months and 12 months post-randomisation

The number of residents who die (from HSCIC data)

**Analysis methods**

**Primary outcome**

The average number of falls occurring during the 3 month period comprising months 4,5 and 6 post randomisation (ie between 91 and 180 days post-randomisation) per participating resident, and the average rate of falls, occurring during the 3 month period comprising months 4, 5 and 6 post randomisation (again number of days spent as resident in the care home between 91 and 180 days post-randomisation, excluding any days spent in hospital during this period), per participating

resident, will be presented by treatment group. Falls rates will be expressed as the number of falls per 1000 participating resident days. The primary effect estimate will be Incidence Rate Ratio, reported with 95% confidence interval.

The number of falls per resident will be compared between groups using a random effects/hierarchical two-level Poisson model with resident at level one and care home at level two, with length of residence in care home as an offset. The primary analysis will adjust for type of care home (residential, nursing, dual registration) and site.

**Adjustment for covariates**

Two further models will adjust for i) baseline fall rate; ii) baseline fall rate and other variables that are associated with falling, in addition to adjusting for care home type and site. Baseline fall rate will comprise information collected on falls in the care home for the 90 days prior to the date of baseline data collection. In the case of participants resident in the care home for fewer than 90 days prior to the baseline data collection, the fall rate will be calculated for the period in which data are available. For residents not fulfilling a minimum period of four weeks of baseline data collection in the care home, missing data will be imputed.

**Methods used for assumptions to be checked for statistical methods**

A negative binomial model will also be fitted, and goodness of fit will be compared between the two models, and the best-fitting model will be reported.

**Details of alternative methods to be used if distributional assumptions do not hold**

If neither Poisson nor negative binomial models are appropriate then alternative models will be explored, or two-stage bootstrap will be used. Alternatively cluster level analyses could be performed.

**Secondary outcomes**

Falls rates for the three month period comprising 7,8 and 9 months post randomisation and for the 3 month period comprising 10, 11 and 12 months post-randomisation will be analysed in a similar way to the primary outcome. Frequency and type of fractures and days in hospital between baseline and 6 months (180 days), and for the period between 6 months (181) and 12 months (360 days).

For other secondary outcomes, groups will be compared using multi-level regression analysis for continuous outcomes (DEMQOL, EQ-5D-5L, medication use, Barthel ADL and PAM-RC) and multi-level logistic regression for binary outcomes (death, at least one fall or not).

**28 Missing data**

A multiple imputation using iteratively chained equations [1] will be used to account for incomplete data and missing data in other outcomes. A random effect will be used to account for the clustering by care-home. The total number of imputations will be approximately the same as the percentage of cases that are incomplete, up to a maximum of 20 imputations. The estimates from imputation will be combined using Rubin's equations. The imputation model used will include all outcome measures and any baseline covariates which are associated with loss-to-follow-up and treatment group.

If any of the variables have a skewed distribution, then transformations will be attempted, however if none are found then predictive mean matching will be used for these variables.

The imputation model may be adapted during the analysis if

a) Perfect prediction is observed. This occurs when the variance-covariance matrix is singular. If this occurs then 'augmenting' will be attempted, but if this does not resolve the issue then variables may be removed from the imputation model.
b) If the imputation procedure does not converge then it will be necessary to remove variables from the imputation model.
c) If the imputation model includes too many variables then instability in the imputation may occur. In this case it may be required to remove variables from the imputation model.

In order to avoid potential issues in model mis-specification, the imputation may not be attempted for outcomes with more than 50% missing data. Multiple imputation will only be used for the intention-to-treat analysis.

**Details of any additional statistical analyses required**
No other analyses are planned at this time

Safety Analyses
No additional analyses will be conducted other than those specified

**Statistical software**
The analysis will be carried out using standard statistical software, either Stata, SAS or R.

**References**[1] White, Ian R., Patrick Royston and Angela M. Wood (2011) "Multiple imputation using chained equations: Issues and guidance for practice." *Statistics in Medicine* 30: 377-399

*Supplementary Table S3: Unit Costs*

| **Description** | **Unit cost UK£ 2017/18** | **Source of Unit cost** |
|---|---|---|
| Ambulance hear & treat or refer | 37 | Reference costs 2017-8 |
| Ambulance see and treat or refer | 192 | Reference costs 2017-8 |
| Advanced clinical practitioner, ANP | 66 | Assume AfC Band 8a |
| Community Mental health team (inc CPN & DOT) | 160 | Ref Costs 2017-8, Other Mental Health Specialist Teams, Adult and Elderly |
| Dentist | 164 | Reference costs 2017-8 |
| Dietician | 86 | Curtis and Burns, PSSRU, 2018 |
| District Nurse (Community matron) | 38 | Reference costs 2017-8 |
| Falls lead | 44 | Assume AfC Band 6 |
| FINCH research staff “Train the Trainer” | 63 | Assume AfC band 8a |
| Any Nurse (phone only) | 19 | Reference costs 2017-8 |
| GP | 34 | Curtis and Burns, PSSRU, 2018 |
| GP (phone only) | 15.1 | Curtis and Burns, PSSRU, 2018 |
| GP (OOH service) | 34 | Curtis and Burns, PSSRU, 2018 |
| Hearing test | 63 | Audiology |
| Home care manager | 40 | Curtis and Burns, PSSRU, 2018 |
| Home care worker | 27 | Curtis and Burns, PSSRU, 2018 |
| Optician | 55 | Assume AfC Band 7 |
| OT | 78 | Curtis and Burns, PSSRU, 2018 |
| Pharmacist | 55 | Assume AfC Band 7 |
| Phlebotomist | 32 | Assume AfC Band 4 |
| Physio & falls team | 54 | Curtis and Burns, PSSRU, 2018 |
| Podiatrist | 41 | Reference costs 2017-8 |
| Practice nurse | 10.5 | Curtis and Burns, PSSRU, 2018 |
| SALT | 97 | Curtis and Burns, PSSRU, 2018 |
| Social worker (adult services) | 84 | Curtis and Burns, PSSRU, 2018 |
| Specialist nurse | 79 | Reference costs 2017-8 |
| GP surgery admin | 8 | Assume AfC Band 4 |
| Social services | 84 | Curtis and Burns, PSSRU, 2018 |
| Support and outreach Worker | 23 | Curtis and Burns, PSSRU, 2018 |
| Funding assessment | 84 | Assume social worker |
| Equipment (Various) | 6.79 to 3,500* | NRS or CCS |
| Medications | Various | Prescription cost analysis, 2018 |

* 1 to 421 at Equivalent Annual Cost

**Supplementary Table S4:** GtaCH intervention costs

| GtACH Intervention costing | *Costing Details/assumptions* | Total | Mean | Min | Max | Component cost per GtACH resident |
|---|---|---|---|---|---|---|
| **# staff trained per CH** | *Taken from GtACH training log: attendees recorded job title & attendance/DNA.* | 1211 | 31.05 | 10 | 70 | 1.53 |
| **# residents recruited per CH** | *Taken from baseline eCRF* | 790 | 20.26 | 10 | 65 | 1.00 |
| **Max # FL Sessions in CH** | *Falls lead travel costs incurred for each session* | 146 | 3.74 | 1 | 13 | 0.18 |
| **Cost components** | | | | | | |
| **Train the Trainer day costs** | *1 day Nottingham; 4 on-site. Includes trial staff time; falls lead time and travel reimbursement* | £11,295 | £289.62 | flat fee per CH | | £14.30 |
| **Training Care Home staff costs** | *Each session costed according to number of staff attending & their roles.* | £31,701 | £812.85 | 283 | 2,025 | £40.13 |
| **Falls lead delivering training to care homes** | *Band 6, 60min training + 30min prep* | £9,636 | £247.08 | 66 | 858 | £12.20 |
| **Falls lead travel to care homes** | *Assume 5 miles, 40p/mile, 30min travel* | £3,504 | £89.85 | 24 | 312 | £4.44 |
| **Cost of consumables** | *2 manuals per care home; 2 Checklists per resident; Attendance certificate per staff member* | £2,381 | £61.04 | | | £3.01 |
| **Undertaking GtACH - CH Staff time** | *Assume undertaken by care home worker (£27/hr) - 30min per participant (NB higher than per protocol - evidence from process evaluation)* | £10,665 | £273.46 | 135 | 877.5 | £13.50 |
| **Residents who fall: Refreshing GtACH – sensitivity analysis** | *Assume if the resident fell, the 30min checklist process (£13.50) would be repeated. Realistic number of refresher sessions – 1 per month – maximum 11 extra sessions (max £14* | £16,861 | £21.34 | 0 (no falls) | 148.5 (11 extra sessio ns) | £21.34 |
| **GtACH cost per Care home (Intervention arm only)** | | £69,182 | £1,773.88 | 858.6 2 | 4530. 62 | |
| | | | | | | |
| **GtACH cost per intervention arm resident** | | | **£87.57** | 53.75 | 208.4 6 | |
| **GtACH cost per resident + refresher** | | | **£108.91** | 53.75 | 356.9 6 | |

**Supplementary Table S5:** Mean (Sd) health and social care costs per resident by intervention group

| | GtACH (n=732) Mean (SD) [no. missing] | Usual Care (n=871) Mean (SD) [no. missing] | Mean Difference (95% CI) (GtACH – Usual) |
|---|---|---|---|
| **Intervention cost without refresher GtACHs** | **88.55 (26.87)** | **0.00 (0.00)** | **88.55 (86.76 to 90.34)** |
| **Intervention cost with refresher GtACHs** | **111.46 (47.44)** | **0.00 (0.00)** | **111.46 (108.29 to 114.62)** |
| **Secondary Care without mortality costs** | **1790.20 (3436.28)** | **1814.74 (3336.26)** | **-24.54 (-357.74 to 308.65)** |
| **Secondary Care including mortality costs** | **1938.87 (3634.96)** | **1927.09 (3459.34)** | **11.78 (-337.02 to 360.58)** |
| Inpatient without mortality costs | 1567.763 (3249.07) | 1581.07 (3148.96) | -13.30 (-328.06 to 301.45) |
| Inpatient including mortality costs | 1716.43 (3447.82) | 1693.42 (3269.41) | 23.02 (-307.21 to 353.25) |
| A&E | 119.67 (207.99) | 126.25 (210.89) | -6.58 (-27.22 to 14.07) |
| Outpatient | 102.76 (255.68) | 107.42 (212.46) | -4.66 (-27.64 to 18.32) |
| **Primary and Community Care** | **728.46 (795.49)** | **646.80 (734.36)** | **81.66 (6.50 to 156.81)** |
| **Equipment** | **17.30 (53.34)** | **20.34 (51.21)** | **-3.036941 - 8.17865 2.104767** |
| **Medications** | **1330.79 (1201.37)** | **1453.66 (1230.13)** | **-122.87 - (242.76 to -2.99)** |
| **Total Cost – Base case** | **3955.29 (3949.38)** | **3935.54 (3879.9)** | **19.76 (-365.88 to 405.39)** |
| **Total Cost - With refresher GtACH** | **3978.2 (3955.87)** | **3935.54 (3879.9)** | **42.66 (-343.32 to 28.64)** |
| **Total Cost - Including extra mortality costs** | **4103.96 (4121.02)** | **4047.89 (3989.66)** | **56.08 (-343.7 to 455.85)** |

| | | | |
|---|---|---|---|
| **Total Cost - Including refresher GtACH and extra mortality costs** | **4126.87 (4127.1)** | **4047.89 (3989.66)** | **78.98 (-321.12 to 479.09)** |

**Supplementary Table S4:** Mean (SD) utility and **QALYs**

| | | GtACH Intervention | | | Usual Care | | |
|---|---|---|---|---|---|---|---|
| | N (max= 732) | Mean | Std dev | N (max= 871) | Mean | Std dev | Mean difference |
| **Proxy Completed** | | | | | | | |
| DEMQOL-P-U baseline | 722 | 0.740 | 0.123 | 866 | 0.737 | 0.124 | 0.003 (-0.009 to 0.015) |
| DEMQOL-P-U 3 months | 699 | 0.655 | 0.248 | 799 | 0.669 | 0.230 | -0.001 (-0.013 to 0.012) |
| DEMQOL-P-U 6 months | 683 | 0.585 | 0.306 | 798 | 0.573 | 0.312 | 0.012 (-0.010 to 0.016) |
| DEMQOL-P-U 9 months | 687 | 0.523 | 0.336 | 801 | 0.524 | 0.346 | -0.001 (-0.006 to 0.010) |
| DEMQOL-P-U 12 months | 666 | 0.477 | 0.357 | 804 | 0.476 | 0.358 | 0.001 (-0.036 to 0.038) |
| **QALYs (DEMQOL-P-U) at 12 months** | **611** | **0.578** | **0.240** | **708** | **0.581** | **0.235** | -0.003 (-0.028 to 0.023) |
| EQ-5D-5L-P baseline | 723 | 0.367 | 0.369 | 867 | 0.344 | 0.360 | 0.021 (-0.015 to 0.057) |
| EQ-5D-5L-P 3 months | 711 | 0.301 | 0.379 | 794 | 0.300 | 0.364 | -0.001 (-0.038 to 0.037) |
| EQ-5D-5L-P 6 months | 702 | 0.260 | 0.361 | 810 | 0.223 | 0.343 | 0.036 (0.000 to 0.072) |
| EQ-5D-5L-P 9 months | 686 | 0.250 | 0.358 | 817 | 0.198 | 0.328 | 0.051 (0.016 to 0.085) |
| EQ-5D-5L-P 12 months | 667 | 0.210 | 0.321 | 803 | 0.162 | 0.313 | 0.047 (0.014 to 0.079) |

| | | | | | | | |
|---|---|---|---|---|---|---|---|
| **QALYs (EQ-5D-5L-P) at 12 months** | **622** | **0.266** | **0.317** | **718** | **0.232** | **0.291** | **0.034 (0.002 to 0.067)** |

Supplementary figure S6: ***Scatter plot and CEAC, DemQol-based QALYs, Primary analysis***

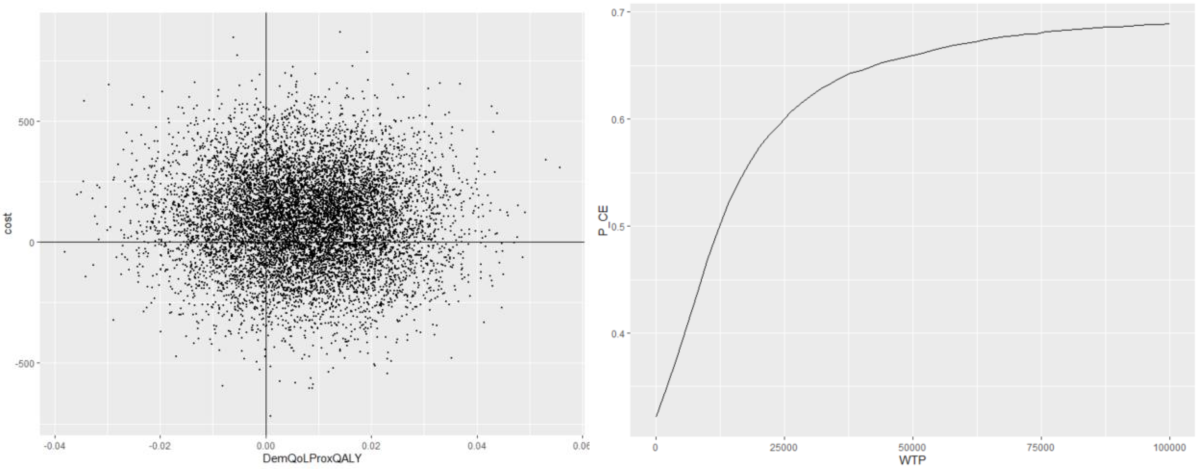


***Supplementary figure S7: Scatter plot and CEAC, EQ5D-based QALYs, Primary analysis***

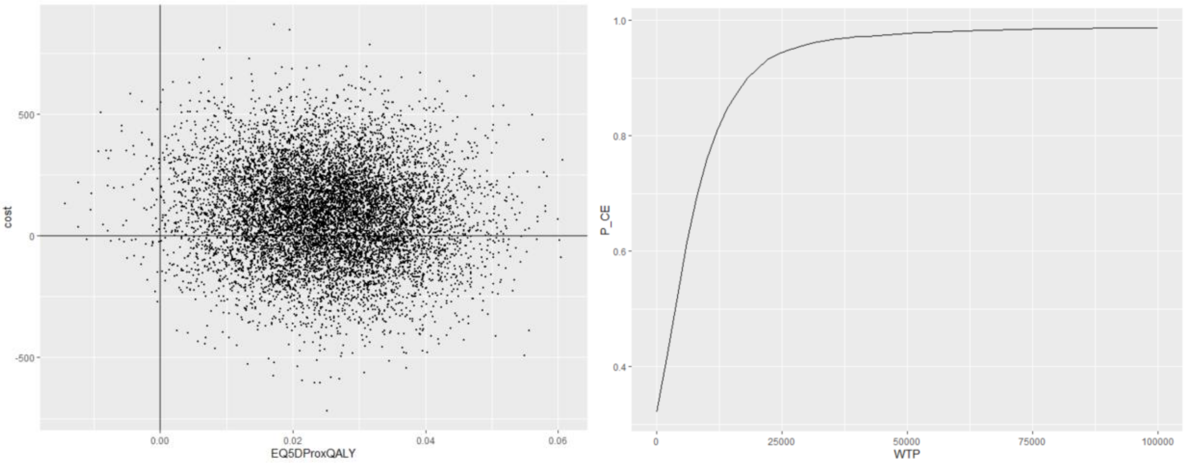


***Supplementary figure S8: Scatterplot and CEAC, falls, Primary analysis (note negative falls = intervention prevents falls)***

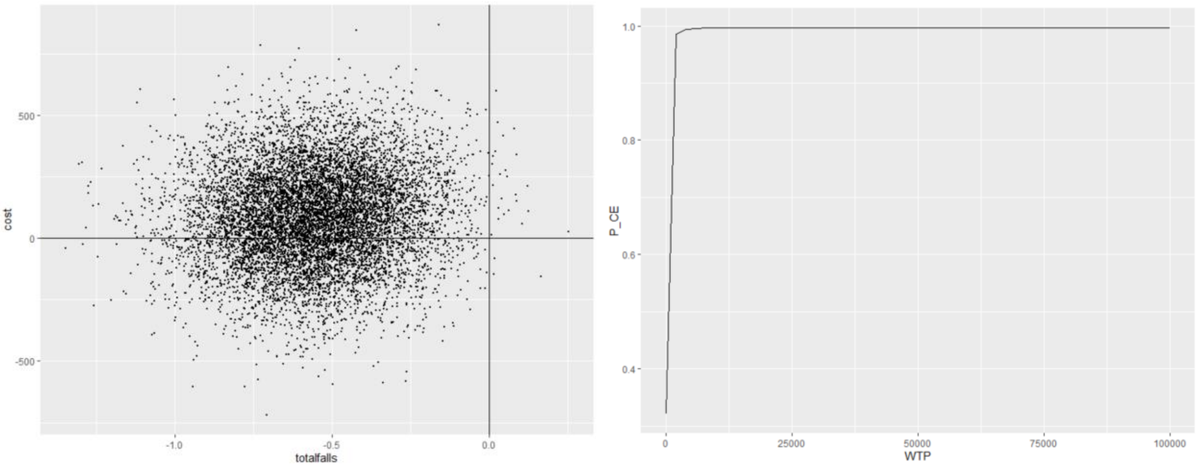

Supplement: Supplementary file 1 — Supplementary information: Additional material [file logp066991.ww.pdf]
